# Um Stent Mal Colocado na Artéria Septal Perfurante: Fístula Ventricular Direita, Hematoma do Septo Interventricular e Obstrução da Via de Saída do Ventrículo Direito

**DOI:** 10.36660/abc.20220901

**Published:** 2023-08-18

**Authors:** Melik Demir, Murat Gök, Muhammet Gürdoğan, Osman Kula, Fethi Emre Ustabaşıoğlu, Kenan Yalta

**Affiliations:** 1 Trakya University Faculty of Medicine Department of Cardiology Edirne Turquia Department of Cardiology Trakya University Faculty of Medicine, Edirne – Turquia; 2 Trakya University Faculty of Medicine Department of Radiology Edirne Turquia Department of Radiology Trakya University Faculty of Medicine, Edirne – Turquia

**Keywords:** Stents, Pontuação de Propensão, Comunicação interventricular/complicações, Fistula Vascular, Obstrução do Fluxo Ventricular, Embolização Terapêutica

## Abstract

As fístulas coronário-camerais, embora consideradas em sua maioria como entidades congênitas, também têm sido encontradas como complicações de grandes traumas e intervenções coronárias percutâneas (ICPs).^1^ Por outro lado, o hematoma do septo interventricular (SIV) pode potencialmente surgir principalmente durante intervenções de oclusão total crônica retrógrada (OTC) e tem um curso benigno nesse contexto.^2^ Aqui, descrevemos uma complicação desafiadora da ICP (e sua estratégia de manejo) apresentando hematoma do SIV, fístula ventricular direita e obstrução da via de saída do ventrículo direito (VSVD) devido a um stent coronário mal implantado na artéria septal perfurante (ASP).

## Caso Clínico

**Figura 1:**
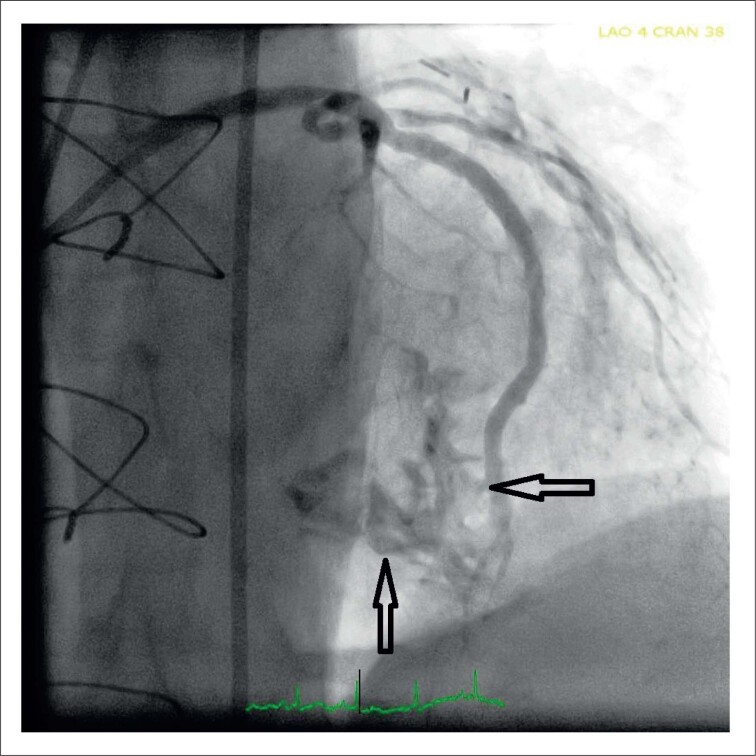
Uma grande fístula drenando para o ventrículo direito.

Um homem de 71 anos foi encaminhado para nossas clínicas de outro centro após uma ICP complicada da artéria descendente anterior (ADA). Registros angiográficos coronarianos (CAG) demonstraram uma má colocação de stent farmacológico (ST) estendendo-se do meio da ADA a uma ASP no SIV. Isso levou a uma grande fístula drenando para o ventrículo direito (VD), possivelmente devido à perfuração da ASP (Figura 1). Também foi implantado enxerto de stent (3,0 X 20 mm), com seu segmento distal sobreposto ao segmento proximal do SF. No entanto, as imagens CAG subsequentes demonstraram a persistência de uma conexão fistulosa.

**Figura 2:**
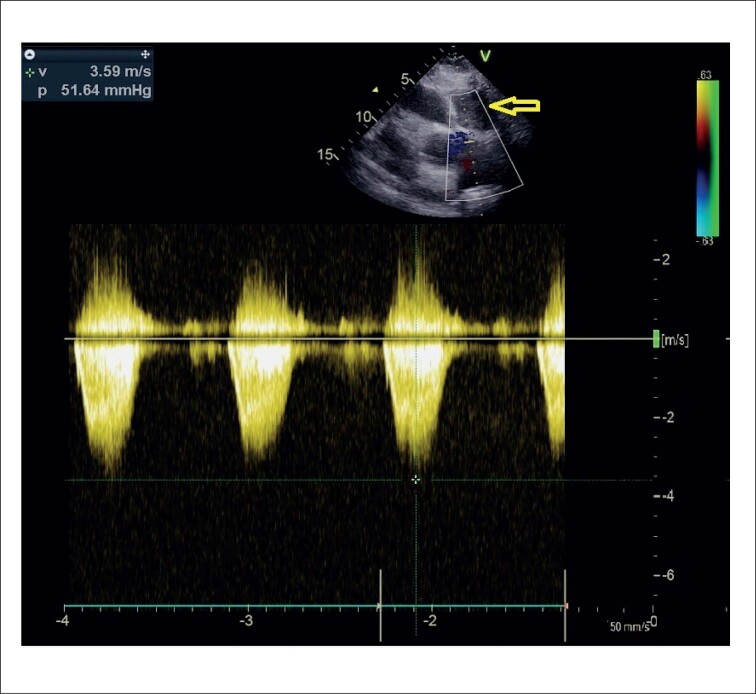
Hematoma subepicárdico comprimindo a via de saída do ventrículo direito na área marcada pela seta e levando a via de saída do ventrículo direito a um gradiente máximo de 50 mmHg.

**Figura 3:**
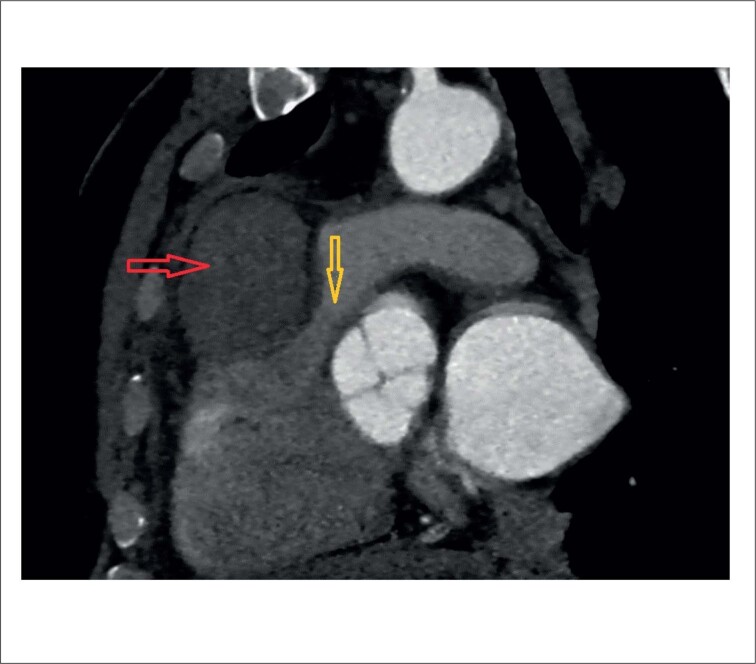
A reconstrução multiplanar da tomografia computadorizada cardíaca representa hematoma subepicárdico, comprimindo e deslocando a via de saída do ventrículo direito. (Seta vermelha: hematoma subepicárdico / Seta amarela: via de saída do ventrículo direito com compressão e estenose).

O ecocardiograma transtorácico (ETT) também demonstrou turbulência colorida contínua que se estendia desde o meio do SIV até a câmara do VD (consistente com fístula). Além disso, uma massa SIV de 50 X 40 mm foi encontrada impactando na via de saída do ventrículo direito (VSVD), levando a um gradiente de pico de 50 mmHg (Figura 2). A tomografia computadorizada (TC) também exibiu sinais de compressão grave da VSVD por uma massa SIV compatível com hematoma (medindo 57 X 40 X 58 mm) (Figura 3).

**Figura 4:**
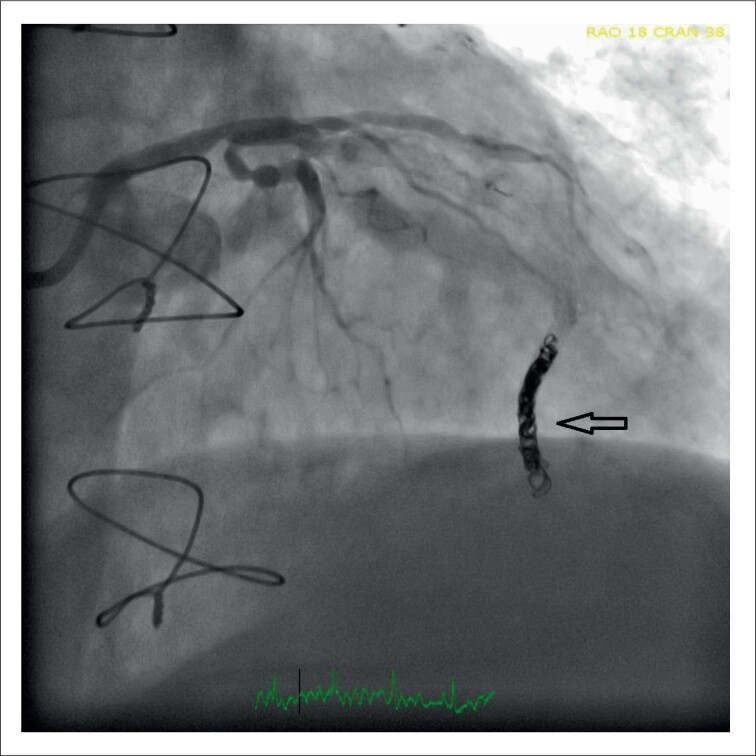
Imagem final das bobinas na artéria descendente anterior (a perfusão distal da artéria descendente anterior foi mantida por colaterais retrógradas).

**Figura 5:**
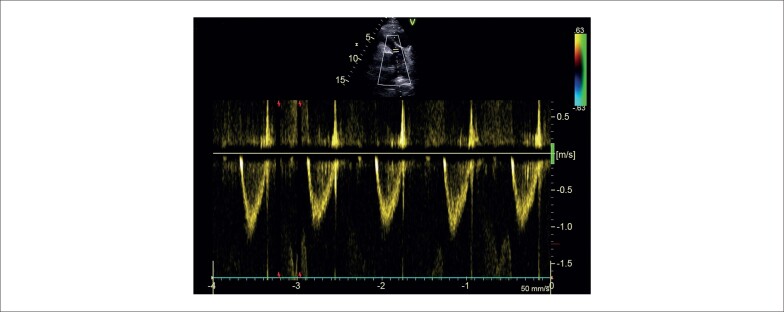
Hematoma septo interventricular e gradiente via de saída do ventrículo direito regrediram significativamente na repetição do ecocardiograma transtorácico.

A conexão fistulosa foi realizada com embolização de molas: 2 molas (Concerto 4x10 cm e 4x8 cm) foram transportadas através de um microcateter (0,18 Asahi) colocado no meio da ADA. A Figura 4 demonstra a imagem final das bobinas na ADA (a perfusão distal da ADA foi mantida por meio de colaterais retrógradas) juntamente com o fechamento completo da drenagem fistulosa (Figura 4). O hematoma SIV e o gradiente VSVD regrediram significativamente no ETT (no seguimento) (Figura 5).

## Discussão

A perfuração iatrogênica da artéria coronária no cenário de ICP tem sido um fenômeno raro.^3^ No entanto, a incidência dessa complicação pode ser relativamente maior na presença de certas características demográficas (idade avançada, sexo feminino) e de procedimento, incluindo morfologia da lesão (lesões calcificadas e tortuosas), intervenções específicas (ICPs para lesões da veia safena e OTCs, uso de dispositivo de aterectomia rotacional) e algumas armadilhas técnicas (relação balão/artéria > 1,2, altas pressões de insuflação, uso de fios-guia rígidos e hidrofílicos).^3–5^ Como esperado, a gravidade da perfuração coronária e o local de drenagem determinam fortemente os desfechos clínicos, inclusive a instabilidade hemodinâmica. Felizmente, as perfurações que se manifestam como fístulas coronário-camerais (em oposição àquelas que drenam para o espaço pericárdico) geralmente são bem toleradas clinicamente. No entanto, não existe consenso sobre o manejo de fístulas coronário-camerais iatrogênicas. Várias estratégias, incluindo insuflação prolongada do balão, embolização de bobina ou tecido adiposo, implante de stent de enxerto e intervenção cirúrgica, foram tentadas,^1–4^ o que pode ser preferido de acordo com as características do paciente e as viabilidades institucionais.

Nesse contexto, uma fístula iatrogênica entre a ADA e a cavidade ventricular esquerda foi relatada anteriormente devido a um fio-guia mal colocado na artéria septal perfurante durante uma ICP anterior e foi tratada com sucesso com implante de stent de enxerto por meio de uma abordagem retrógrada.^1^ Em outro estudo anterior, caso submetido a intervenção OTC retrógrada, hematoma do SIV emergente e fístula de VD foram manejados com implante de stent de enxerto e embolização de molas.^2^ No presente caso, o implante de stent foi a estratégia de conduta inicial. No entanto, o stent do enxerto não conseguiu terminar a conexão fistulosa, possivelmente devido a fatores como falha geográfica, múltiplos locais de perfuração ou perfuração na ponta distal do ASP. Portanto, usamos a embolização da bobina como o próximo passo e terminamos com sucesso a conexão fistulosa à cavidade do VD.

Outro aspecto particular do presente caso foi o hematoma SIV emergente (associado a um gradiente significativo da VSVD) que regrediu no seguimento. Com base no consenso geral,^2,3^ não realizamos nenhuma intervenção cirúrgica como estratégia inicial para esse hematoma devido à ausência de características de alto risco, incluindo comprometimento hemodinâmico e aumento progressivo. Tomados em conjunto, o principal fator associado a essas complicações parece ser o mau posicionamento do fio-guia no paciente. Portanto, a avaliação de múltiplas imagens angiográficas (e injeção de ponta por meio de um microcateter) parece ser uma estratégia razoável para a colocação adequada do fio-guia^1^ e posterior implantação do stent, principalmente durante a ICP de oclusões coronárias totais.

## Conclusão

Fístulas coronário-camerais iatrogênicas e hematomas SIV raramente foram encontrados em pacientes submetidos a ICP, particularmente aqueles com características anatômicas e de procedimentos de alto risco. No entanto, o surgimento dessas complicações também pode ser possível mesmo no cenário de intervenções coronarianas relativamente simples (nas configurações de OTC anterógrada ou mesmo sem OTC). Portanto, todos os esforços devem ser feitos para prevenir e gerenciar oportunamente essas complicações. Notavelmente, as estratégias de manejo devem ser implementadas caso a caso.
