# Supplementary material for: Um Stent Mal Colocado na Artéria Septal Perfurante: Fístula Ventricular Direita, Hematoma do Septo Interventricular e Obstrução da Via de Saída do Ventrículo Direito
Source: Arq Bras Cardiol. 2023 Aug 18;120(8):e20220901. [Article in Portuguese] doi: 10.36660/abc.20220901 (PMC10519249; doi:10.36660/abc.20220901)
Supplement: Translation

# Translation: A Stent Misplaced in the Septal Perforating Artery: Right Ventricular Fistula, Interventricular Septal Hematoma, and Right Ventricular Outflow Tract Obstruction

**Authors:** Melik Demir, Murat Gök, Muhammet Gürdoğan, Osman Kula, Fethi Emre Ustabaşıoğlu, Kenan Yalta

**Affiliations:** 1 Edirne Turkey Department of Cardiology Trakya University Faculty of Medicine, Edirne – Turkey; 2 Edirne Turkey Department of Radiology Trakya University Faculty of Medicine, Edirne – Turkey

**Keywords:** Stents, Propensity Score, Heart Septal Ventricular/complications, Vascular Foistula, Ventricular Outflow Obstruction, Embolization Therapeutc

## Abstract

Coronary-cameral fistulas, though mostly regarded as congenital entities, have also been encountered as complications of major traumas and percutaneous coronary interventions (PCIs).^1^ On the other hand, interventricular septal (IVS) hematoma might potentially arise mostly during retrograde chronic total occlusion (CTO) interventions and has a benign course in this context.^2^ Herein, we describe a challenging PCI complication (and its management strategy) presenting with IVS hematoma, right ventricular fistula, and right ventricular outflow tract (RVOT) obstruction due to a misimplanted coronary stent in the septal perforating artery (SPA).

## Clinical case

A 71-year-old male was referred to our clinics from another center following a complicated PCI of the left anterior descending artery (LAD). Coronary angiographic (CAG) records demonstrated a misplacement of drug-eluting stent (DES) extending from the mid-LAD to a SPA in the IVS. This led to a large fistula draining into the right ventricle (RV), possibly due to the perforation of SPA (Figure 1). A graft stent (3.0 X 20 mm) was also implanted, with its distal segment overlapping with the proximal segment of the DES. However, subsequent CAG images demonstrated the persistence of a fistulous connection.

**Figure 1:** A large fistula draining into the right ventricle.

Transthoracic echocardiogram (TTE) also demonstrated continuous color turbulence that extended from the mid-IVS to the RV chamber (consistent with fistula). Moreover, an IVS mass of 50 X 40 mm was found to impinge on the right ventricular outflow tract (RVOT), leading to a peak gradient of 50 mmHg (Figure 2). Computed tomography (CT) also exhibited signs of severe RVOT impingement by an IVS mass consistent with hematoma (measuring 57 X 40 X 58 mm) (Figure 3).

**Figure 2:** Subepicardial hematoma compressing the right ventricular outflow tract in the arrow-marked area and right ventricular outflow tract leading to a peak gradient of 50 mmHg.

**Figure 3:** Cardiac computed tomography multiplanar reformation represents subepicardial hematoma, compressing and displacing the right ventricular outflow tract. (Red arrow: subepicardial hematoma / Yellow arrow: right ventricular outflow tract with compression and stenosis).

The fistulous connection was managed with coil embolization: 2 coils (Concerto 4x10 cm ve 4x8 cm) were transported through a microcatheter (0.18 Asahi) placed in mid-LAD. Figure-4 demonstrates the final image of coils in the LAD (distal LAD perfusion was maintained via retrograde collaterals) along with the complete closure of the fistulous drainage (Figure 4). IVS hematoma and RVOT gradient regressed significantly on TTE (on follow-up) (Figure 5).

**Figure 4:** Final image of coils in the left anterior descending artery (distal left anterior descending artery perfusion was maintained via retrograde collaterals).

**Figure 5:** Interventricular septal hematoma and right ventricular outflow tract gradient regressed significantly on repeat transthoracic echocardiogram.

## Discussion

Iatrogenic coronary artery perforation in the setting of PCI has been a rare phenomenon.^3^ However, the incidence of this complication may be relatively higher in the presence of certain demographic (advanced age, female gender) and procedural features, including lesion morphology (calcified and tortuous lesions), specific interventions (PCIs for saphenous vein lesions, and CTOs, use of rotational atherectomy device) and certain technical pitfalls (balloon /artery ratio > 1.2, high inflation pressures, use of stiff and hydrophilic guidewires).^3–5^ As expected, the severity of coronary perforation and drainage site strongly determine the clinical outcomes, including hemodynamic instability. Fortunately, perforations manifesting as coronary-cameral fistulas (as opposed to those draining to the pericardial space) are generally well tolerated clinically. However, there exists no consensus on the management of iatrogenic coronary-cameral fistulas. Various strategies, including prolonged balloon inflation, coil or fat tissue embolization, graft stent implantation, and surgical intervention, have been tried,^1–4^ which may be preferred according to patient characteristics and institutional feasibilities.

In this context, an iatrogenic fistula between the LAD and left ventricular cavity was previously reported to occur due to a misplaced guidewire in the septal perforating artery during a previous PCI and was successfully managed with graft stent implantation through a retrograde approach.^1^ In another previous case undergoing retrograde CTO intervention, emerging interventricular septal hematoma and RV fistula were managed with graft stent implantation and coil embolization.^2^ In the present case, graft stent implantation was the initial management strategy. However, the graft stent failed to terminate the fistulous connection, possibly due to factors such as geographic miss, multiple perforation sites, or perforation at the distal tip of the SPA. Therefore, we used coil embolization as the next step and successfully terminated the fistulous connection to the RV cavity.

Another particular aspect of the present case was the emerging IVS hematoma (associated with a significant RVOT gradient) that regressed on follow-up. Based on general consensus,^2,3^ we did not undertake any surgical intervention as the initial strategy for this hematoma due to the absence of high-risk features, including hemodynamic compromise and progressive enlargement. Taken together, the primary factor associated with these complications seem to be the misplacement of the guidewire in the patient. Therefore, evaluating multiple angiographic images (and tip injection through a microcatheter) seems to be a reasonable strategy for proper guidewire placement^1^ and subsequent stent implantation, particularly during PCI of total coronary occlusions.

## Conclusion

Iatrogenic coronary-cameral fistulas and IVS hematomas have rarely been encountered in patients undergoing PCI, particularly those with high-risk anatomical and procedural features. However, the emergence of these complications may also be possible even in the setting of relatively simple coronary interventions (in the antegrade CTO or even non-CTO settings). Therefore, every effort should be made to prevent and timely manage these complications. Notably, management strategies should be implemented on a case-by-case basis.
